# Effects of Zinc Toxicity on Chlorophyll Fluorescence and Translocation Factor of *Potentilla chinensis*

**DOI:** 10.3390/life16091562

**Published:** 2026-09-17

**Authors:** Yue Wang, Yu Qiao, Sen Yang, Weizhou Xu, Yaojun Bo, Xia Han

**Affiliations:** College of Advanced Agricultural Sciences, Shaanxi Engineering Research Center of Forage Plants of the Loess Plateau, Yulin University, Yulin 719000, China; wyue1102@126.com (Y.W.);

**Keywords:** *Potentilla chinensis*, zinc (Zn), chlorophyll fluorescence, phytoremediation, root parameters

## Abstract

Zinc (Zn) is essential for plant metabolism but becomes toxic at high concentrations. In this study, we investigated the effects of Zn concentrations (40, 80, 160, 240, 320, 400 mg L^−1^) on plant growth, root morphology, chlorophyll fluorescence, and Zn accumulation in *Potentilla chinensis* grown in soil for 21 days. The results showed that total root length, root surface area, root volume, qP, qL, ETR, and SPAD values responded at ≤240 mg L^−1^, whereas plant height was affected at 400 mg L^−1^, indicating that SPAD, some fluorescence, and root traits responded before major biomass effects became statistically significant. At 400 mg L^−1^, shoot and root Zn concentrations reached 1353.75 and 187.16 mg kg^−1^ DW, respectively, and the translocation factor was 7.24. F_m_, F_v_, F_v_/F_m_, and ΦPSII remained stable, whereas F_0_ decreased and NPQ increased, indicating that PSII function was largely maintained, with partial reaction center damage offset by enhanced protective thermal dissipation. Shoot Zn concentrations exceeded root Zn concentrations across all treatments, indicating efficient root-to-shoot Zn translocation. These findings show that *P. chinensis* exhibits Zn accumulation and efficient root-to-shoot translocation under the tested conditions and warrants further investigation as a candidate species for Zn phytoextraction.

## 1. Introduction

Zinc (Zn) is not only a component of more than 300 enzymes but also a micronutrient essential for normal plant growth and development [1]. It participates in many physiological and biochemical processes, including respiration, photosynthesis, and hormone biosynthesis, and its effects on photosynthetic performance can be evaluated using chlorophyll fluorescence parameters [2]. Ordos in the Inner Mongolia Autonomous Region and Yulin in Shaanxi Province were major mining regions with abundant coal and lead–zinc deposits. They are in the Mu Us Sandy Land. Long-term mining has caused metal contamination in parts of these regions, posing risks to the fragile local ecosystem [3,4]. When Zn concentrations in soil or growth media exceed the threshold for normal plant growth, they can inhibit growth and even cause phytotoxicity [5,6]. Although the toxicity threshold for Zn varies widely among plant species, there might also be significant differences among individuals of the same species across different habitats [7]. Most commonly, the toxic concentration range of Zn in various plants is 100–500 mg kg^−1^ dry weight (DW), with toxic effects manifesting as stunted plant growth and leaf chlorosis [8]. This toxic effect causes chlorosis in plant leaves, nutritional imbalances, and impaired photosynthesis, thereby affecting plant growth [9,10].

Phytoremediation is an environmentally friendly remediation strategy that used plants to remove, sequester, translocate, or stabilize contaminants, and it has become an important approach for remediating metal-contaminated soils [11,12,13]. Plant tolerance to metals, together with their capacity for metal uptake, root-to-shoot translocation, and accumulation, is critical in determining their phytoremediation potential [14,15]. The juvenile stage is a critical period for root system establishment and the development of photosynthetic capacity, during which plants were particularly sensitive to metal stress [16]. Huang et al. [17] found that Zn may inhibit the activity of photosystem II and the Calvin cycle by inhibiting grana formation and electron transfer, thereby ultimately inhibiting photosynthesis in *Koelreuteria paniculata* and *Zelkova schneideriana*. Studies on *Phaseolus vulgaris* and *Bacopa monnieri* have shown that high Zn exposure causes leaf Zn accumulation, which inhibits photosynthesis by disrupting chloroplast electron transport and CO_2_ fixation [18,19].

*Potentilla chinensis* Ser. is a perennial herbaceous species in the genus *Potentilla* of the family Rosaceae and was one of the dominant wild perennial herbs in the Mu Us Sandy Land of China [20]. A previous study of two perennial forbs on the Loess Plateau, alfalfa and *P. chinensis*, examined the relationship between root xylem annual ring width and climatic factors and found that *P. chinensis* maintained relatively stable growth under drought, nutrient-poor, and sandy conditions [21], indicating its ecological adaptability to arid and semi-arid grassland environments. Wang et al. [22] found that *P. chinensis* showed potential to accumulate multiple heavy metals, as indicated by its biomass, bioconcentration factor, translocation factor (TF), and metal extraction rate. At present, the effects of Zn stress on plant growth, root morphology, chlorophyll fluorescence, and Zn accumulation and translocation capacity in *P. chinensis* have been reported. As the first organ to sense Zn stress, the root system absorbs Zn and translocates it to the shoots, where it impairs photosynthetic performance and ultimately manifests as plant growth. However, systematic integration of these indicators to explain the impact of Zn on plant growth remains limited. Therefore, this experiment aimed to clarify the effects of different Zn concentrations on the root morphology of *P. chinensis*, its characteristics of Zn accumulation and translocation, the impact of shoot Zn enrichment on PSII photochemical efficiency, and the sequence of phenotypic responses by measuring plant growth, root architecture, chlorophyll fluorescence, and Zn accumulation and translocation coefficients under gradient Zn treatments. The findings provide a basis for further evaluating *P. chinensis* as a native candidate for phytoremediation of Zn-contaminated sites in mining areas.

## 2. Materials and Methods

### 2.1. Plant Material, Growth Conditions, and Treatments

**Figure 1:**
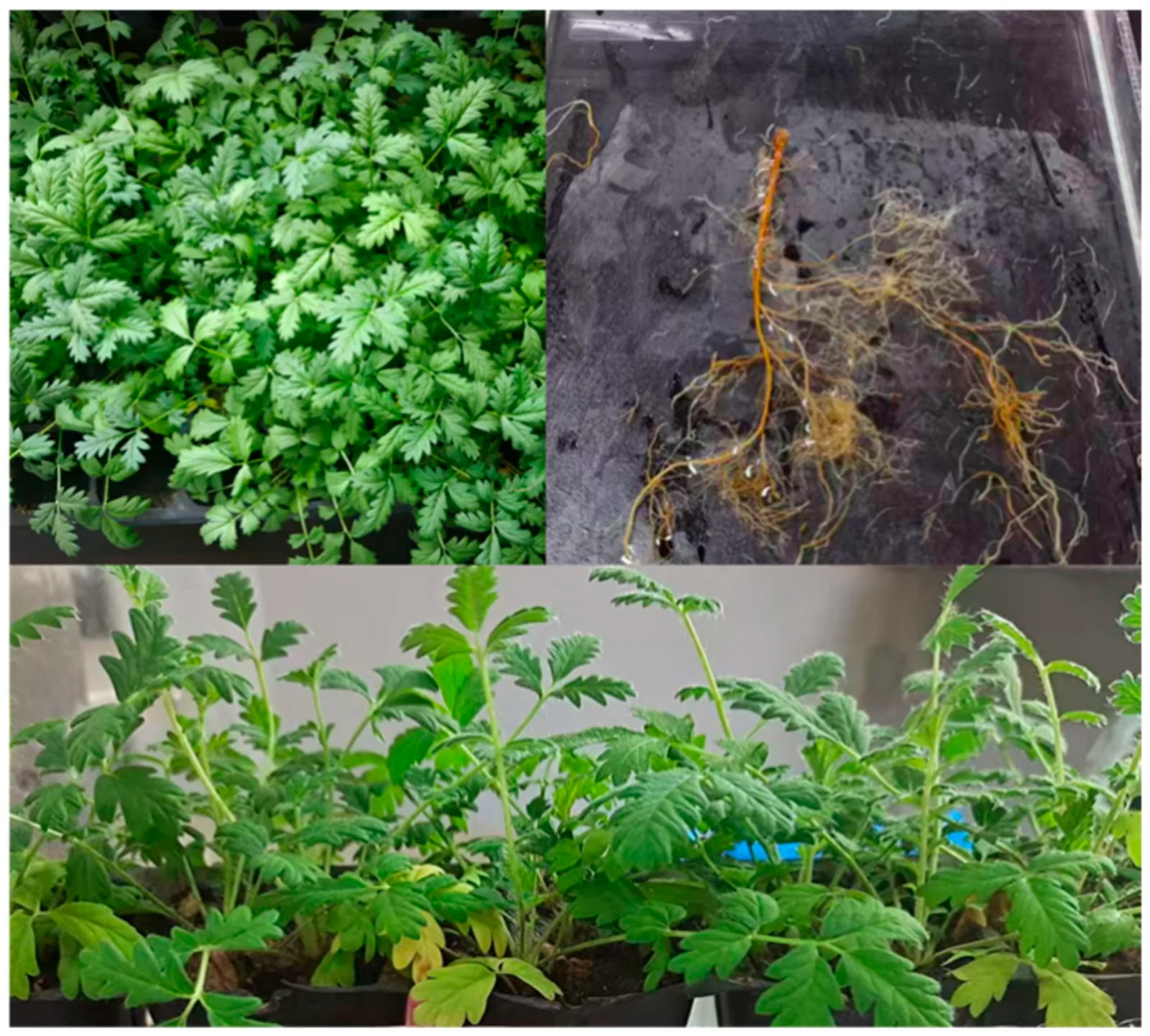
*Potentilla chinensis*.

Seeds of *P. chinensis* were collected in September 2024 from Tuke Town, Ordos City, Inner Mongolia Autonomous Region. Select plump, intact seeds free of pests and diseases, and sow them on 25 January 2025 in plug trays with 9 cm-deep cells. Each tray was placed in a matching bottom tray to prevent leachate loss. The growing medium was commercially available potting soil, primarily composed of peat, vermiculite, perlite, and coconut coir. After germination, the seeds were grown under standard conditions for 95 days, by which time each compound leaf of *Potentilla chinensis* had 3–5 pairs of leaflets and had not yet begun to form flower buds (Figure 1). According to the Soil Environmental Quality—Risk Control Standard for Soil Contamination of Agricultural Land (GB 15618–2018) [23] and relevant literature, Zn treatments were set at 0 (control, CK), 40, 80, 160, 240, 320, and 400 mg L^−1^, with three independent biological replicates per treatment. Each biological replicate consisted of an independent plant. Zn was added in the form of ZnSO_4_·7H_2_O. During the 21-day stress treatment, plants were irrigated with 25 mL of the corresponding ZnSO_4_·7H_2_O solution every 5 days. The pH of the Zn treatment solutions was adjusted to approximately 5.6 before application. The total amount of Zn applied per treatment during the experiment was 0, 5, 10, 20, 30, 40, and 50 mg, respectively.

### 2.2. Measurement of Growth Parameters

Three *P. chinensis* plants were selected per treatment. Plant height was measured from the stem base to the shoot apex with a ruler. The plants were divided into shoots and roots. The samples were first heated at 105 °C for 30 min and then dried at 75 °C to constant weight to determine shoot and root DW. The root-to-shoot ratio was calculated as the root DW divided by the shoot DW.

### 2.3. Measurement of Root Morphological Parameters

The root systems of *P. chinensis* were scanned using aPerfection V850 Pro flatbed scanner (Seiko Epson Corporation, Suwa, Japan) to obtain digital images, which were analyzed with WinRHIZO Pro 2015 software to determine total root length, root surface area, root volume, and root diameter.

### 2.4. Measurement of SPAD Values

Three *P. chinensis* plants were selected per treatment, and three mature leaves were randomly chosen from each plant. SPAD values readings were taken at three points on each leaf using a handheld chlorophyll meter (SPAD-502), avoiding the midrib, and the mean was calculated.

### 2.5. Measurement of Chlorophyll Fluorescence Parameters

Mature leaves were collected from three *P. chinensis* plants per treatment, and chlorophyll fluorescence parameters were measured with a portable pulse-amplitude-modulated chlorophyll fluorometer (PAM-2500, Heinz Walz GmbH, Effeltrich, Germany). Measurements were conducted around midday under natural daylight conditions. Leaves were dark-adapted for 30 min using dark-adaptation clips before the minimum fluorescence (F_0_) and maximum fluorescence (F_m_) were measured using a saturation pulse (intensity level 10, duration 50 ms). Leaves were dark-adapted for 30 min before the minimum fluorescence (F_0_) and maximum fluorescence (F_m_) were measured. Plants were then light-adapted for 30 min, after which the effective quantum yield of PSII photochemistry (ΦPSII), non-photochemical quenching (NPQ), photochemical quenching (qP), fraction of open PSII reaction centers (qL), electron transport rate (ETR), and maximum quantum efficiency of PSII photochemistry (F_v_/F_m_) were recorded. Variable fluorescence (F_v_) was calculated as follows:

F_v_ = F_m_ − F_0_

### 2.6. Determination of Zn Concentrations and Translocation Factor

The shoot and root samples were oven-dried, ground, and passed through a 0.28 mm sieve. Approximately 1.00 g of dried samples were accurately weighed and digested with 15 mL of a nitric acid-perchloric acid mixture. After digestion, the solution was filtered and diluted to a final volume of 25 mL, and a reagent blank was prepared simultaneously. The total Zn content in plant samples was determined using atomic absorption spectrophotometry (ASS, Z-2000, Hitachi, Japan). A Zn standard stock solution (100 μg mL^−1^) was prepared using pure metallic Zn, and a series of Zn standard solutions (0, 0.2, 0.4, 0.6, 0.8, and 1.0 μg mL^−1^) was prepared to establish the calibration curve. The Zn content was calculated according to the regression equation obtained from the calibration curve. The Zn content was calculated using the following equation:

Zn content (mg·kg^−1^) = [(ρ − ρ_0_) × V]/m

where ρ represents the Zn concentration in the sample solution (μg·mL^−1^), ρ_0_ represents the Zn concentration in the blank solution (μg·mL^−1^), V represents the final volume of the digestion solution (mL), and m represents the dry weight of the plant sample (g).

The Zn translocation factor (TF) was calculated as the ratio of shoot Zn concentration to root Zn concentration. Whole-plant Zn concentration was calculated as the dry-weighted mean of shoot and root Zn concentrations:

C_whole_ = (C_shoot_ × M_shoot_ + C_root_ × M_root_)/(M_shoot_ + M_root_)

where C_whole_ is the whole-plant Zn concentration, C_shoot_ and C_root_ are the Zn concentrations in shoots and roots, respectively, and M_shoot_ and M_root_ are the dry weights of shoots and roots, respectively.

### 2.7. Statistical Analysis

Data were organized in Microsoft Excel 2023 and analyzed in IBM SPSS Statistics 27.0. Results are presented as mean ± standard deviation (SD). Treatment effects were assessed using one-way analysis of variance (ANOVA) followed by Duncan’s multiple range test, with significance set at *p* < 0.05 or *p* < 0.01. Statistical analyses were performed using the three biological replicates as independent experimental units. Pearson’s correlation analysis was performed to assess the relationships among growth, root morphological, SPAD, and chlorophyll fluorescence parameters. Figures were prepared in Origin 2021.

## 3. Results

### 3.1. Growth Performance of P. chinensis Under Zn Stress

Under Zn stress, shoot and root DW and plant height of *P. chinensis* decreased with increasing Zn concentration, and the root-to-shoot ratio increased. However, only plant height differed significantly from the CK (*p* < 0.05) (Table 1). Compared with the CK, plant height decreased significantly by 21.00% under the 400 mg L^−1^ Zn treatment (*p* < 0.05). No significant differences in plant height were observed at other zinc concentrations (*p* > 0.05). At 400 mg L^−1^ Zn, root and shoot DW showed numerical decreases of 20.00% and 31.58%, respectively, and the root-to-shoot ratio showed a numerical increase of 10.71%; however, these changes were merely numerical trends and did not reach statistical significance (*p* > 0.05).

As Zn concentration increased, the total root length, root surface area, and root volume of *P. chinensis* initially increased and then decreased. The maximum values for total root length and root surface area were observed at 40 mg L^−1^ Zn treatment, while root volume reached its peak at 160 mg L^−1^ Zn treatment; all three parameters significantly declined at 240 mg L^−1^ Zn treatment. Specific root length and root diameter showed no significant differences among treatments (Table 2). Total root length and root surface area reached their maximum values at 40 mg L^−1^ Zn, at 426.56 cm and 23.81 cm^2^, respectively, representing significant increases of 5.39% and 2.54% compared with the CK (*p* < 0.05). However, there was no significant difference between these two parameters at Zn concentrations of 80 and 160 mg L^−1^ (*p* > 0.05). Compared with the CK, total root length was significantly reduced by 41.20%, 40.40%, and 42.27% under 240, 320, and 400 mg L^−1^ Zn treatments, respectively (*p* < 0.05). Root surface area showed significant decreases of 20.54%, 19.81%, and 21.79% (*p* < 0.05). Root volume increased significantly by 58.33% and 91.67% under 40 and 160 mg L^−1^ Zn treatments, respectively, but decreased by 29.17%, 45.83%, and 70.83% under 240, 320, and 400 mg L^−1^ Zn treatments, respectively (*p* < 0.05). However, specific root length and root diameter did not differ significantly from the CK across all Zn treatments (*p* > 0.05). In summary, Zn concentrations below 160 mg L^−1^ appeared to promote root growth in *P. chinensis*, whereas concentrations above 240 mg L^−1^ significantly inhibited it.

### 3.2. Effects of Zn Stress on SPAD Values in P. chinensis

**Figure 2:**
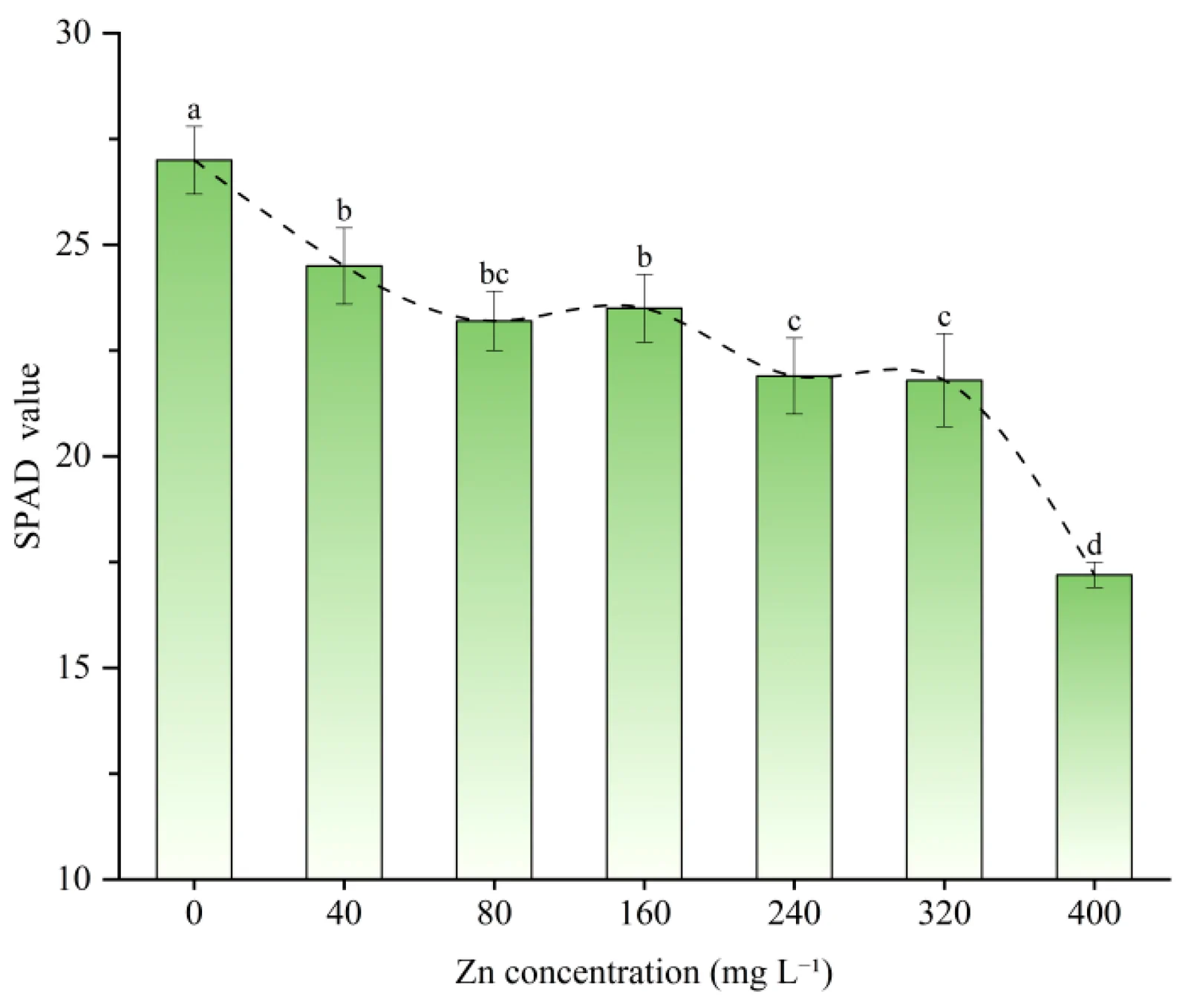
Effects of different Zn treatments on SPAD values of *Potentilla chinensis.* Data are presented as mean ± SD (*n* = 3). Different lowercase letters indicate significant differences among treatments according to Duncan’s multiple range test (*p* < 0.05).

As Zn concentration increased, SPAD values in *P. chinensis* significantly decreased (*p* < 0.05) (Figure 2). Compared with the CK, SPAD values decreased significantly by 9.26%, 14.07%, 12.96%, 18.89%, 19.26%, and 36.30% at Zn concentrations of 40, 80, 160, 240, 320, and 400 mg L^−1^, respectively (*p* < 0.05). These results indicate that SPAD values, as an indirect indicator of leaf pigment status, were sensitive to Zn stress and declined progressively with increasing Zn concentration.

### 3.3. Effects of Zn Stress on Chlorophyll Fluorescence Parameters of P. chinensis

**Figure 3:**
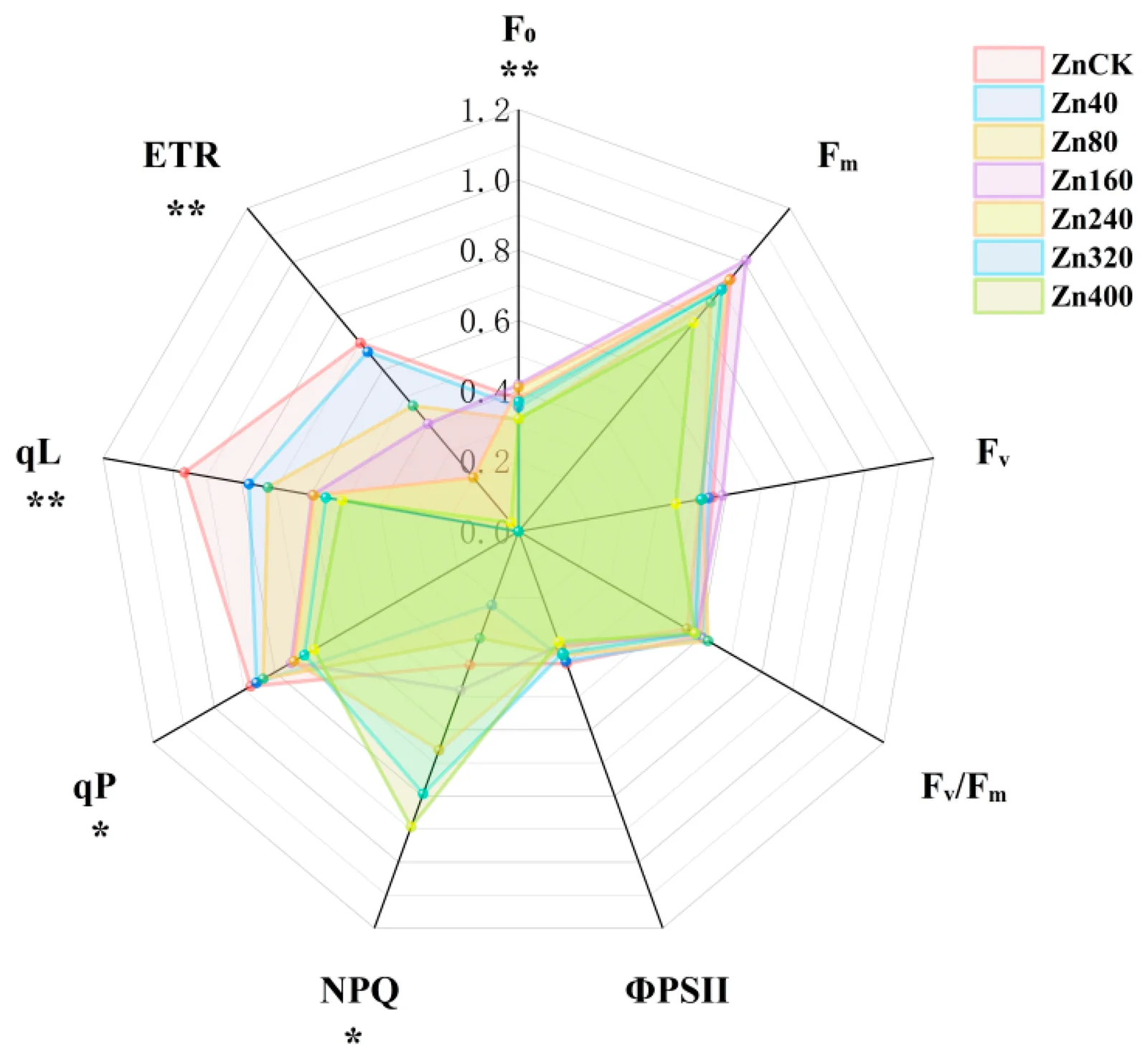
Effects of Zn treatments on chlorophyll fluorescence parameters of *Potentilla chinensis.* Values represent treatment means (*n* = 3). Asterisks indicate significant differences compared with the control (* *p* < 0.05; ** *p* < 0.01).

Under Zn stress, F_m_, F_v_, F_v_/F_m_, and ΦPSII in *P. chinensis* did not differ significantly from those of the CK (*p* > 0.05). As Zn concentration increased, NPQ values increased significantly (*p* < 0.05); qP values decreased significantly (*p* < 0.05); ETR and qL values decreased significantly (*p* < 0.01); F_0_ initially increased and then decreased (*p* < 0.01) (Figure 3). Compared to the CK, qP values decreased significantly by 15.11% at 160 mg L^−1^ (*p* < 0.05); qL values decreased significantly by 19.27% at 40 mg L^−1^ (*p* < 0.01); and ETR values decreased significantly by 42.86% at 160 mg L^−1^ (*p* < 0.01). NPQ values increased significantly by 54.81% compared to the CK at 400 mg L^−1^ (*p* < 0.05). F_0_ values decreased by 15.61% and 16.08% at Zn concentrations of 80 and 400 mg L^−1^, respectively (*p* < 0.01). There was no significant difference between the two treatments (*p* > 0.01). F_0_ in the remaining treatments did not differ significantly from CK (*p* > 0.01).

### 3.4. Effects of Zn Stress on Zn Content and Translocation Factor in P. chinensis

**Figure 4:**
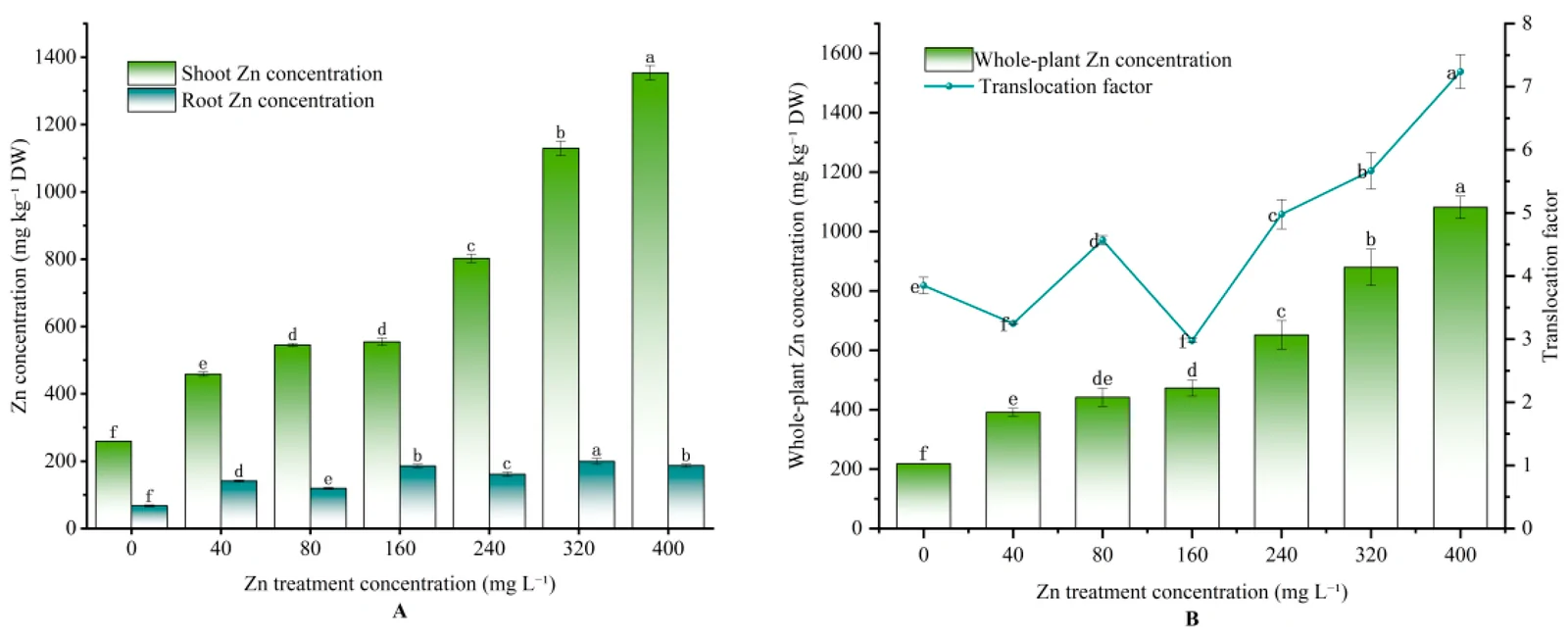
Effects of different Zn treatments on Zn accumulation and translocation in *Potentilla chinensis*. (**A**) Zn concentrations in shoots and roots. (**B**) Whole-plant Zn concentration and translocation factor. Values are presented as means ± SD (*n* = 3). Different lowercase letters indicate significant differences among treatments at *p* < 0.05.

As Zn concentration increased, Zn concentrations in the shoots, roots, and whole plants of *P. chinensis* increased significantly, whereas the Zn translocation factor showed a W-shaped pattern (Figure 4). The shoot parts of *P. chinensis* were the main site of Zn enrichment, demonstrating significant Zn retention capacity (Figure 4A). Compared with the CK (259.20 mg kg^−1^ DW), shoot Zn concentrations increased progressively to 458.57, 545.35, 554.57, 802.46, 1129.82, and 1353.75 mg kg^−1^ DW at Zn concentrations of 40, 80, 160, 240, 320, and 400 mg L^−1^, respectively (*p* < 0.05). The shoot Zn accumulation in the 400 mg L^−1^ treatment was 5.22 times that of the CK. An increase in Zn concentration significantly enhanced Zn accumulation in roots, although the increase was smaller compared to shoot parts. The root Zn content ranged from 67.4 to 199.48 mg kg^−1^ DW. As shown in Figure 4B, the TF generally increased with increasing Zn concentration. At 40 and 160 mg L^−1^, the translocation factor (TF) was significantly lower than that of the control, whereas the remaining treatments were significantly higher than that of the control (*p* < 0.05). The TF reached a maximum of 7.24 at the 400 mg L^−1^ treatment and a minimum of 2.98 at the 160 mg L^−1^ treatment.

### 3.5. Correlation Analysis of Growth and Physiological Traits in P. chinensis Under Zn Stress

**Figure 5:**
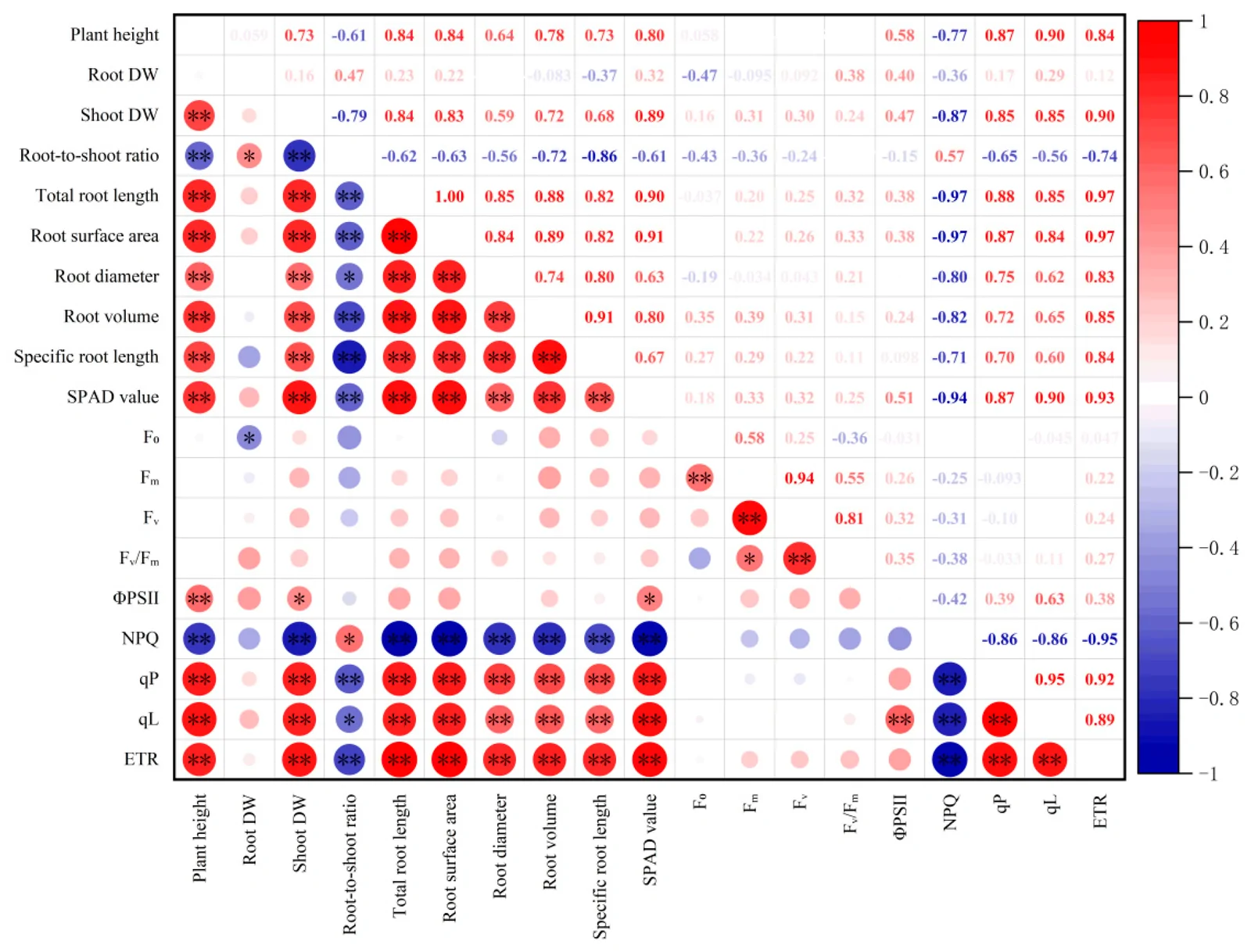
Pearson correlation analysis of growth and physiological parameters of *Potentilla chinensis* under different Zn treatments. The correlation coefficients are shown in the matrix, with red and blue representing positive and negative correlations, respectively. The color intensity indicates the strength of the correlations. Asterisks indicate significant correlations (* *p* < 0.05; ** *p* < 0.01).

Zn stress significantly affected the growth and physiological parameters of *P. chinensis*, and there was a complex correlation between growth and physiological indices (Figure 5). Root DW was significantly positively correlated with the root-to-shoot ratio and significantly negatively correlated with F_0_ (*p* < 0.05). Both plant height and shoot DW were significantly positively correlated with total root length, root surface area, root diameter, root volume, specific root length, SPAD values, qP, qL, and ETR, and significantly negatively correlated with the root-to-shoot ratio and NPQ (*p* < 0.01). Additionally, plant height was significantly positively correlated with shoot DW and ΦPSII (*p* < 0.01), whereas shoot DW showed only a significant positive correlation with ΦPSII (*p* < 0.05). The root-to-shoot ratio showed a significant positive correlation with NPQ (*p* < 0.05), significant negative correlations with total root length, root surface area, root volume, specific root length, SPAD values, qP, and ETR (*p* < 0.01), and significant negative correlations with root diameter and qL (*p* < 0.05). Total root length, root surface area, root diameter, root volume, specific root length, and SPAD values were all significantly and negatively correlated with NPQ (*p* < 0.01). The following parameters were significantly correlated with each other: total root length with root surface area, root diameter, root volume, specific root length, SPAD values, qP, qL, and ETR (*p* < 0.01); root surface area with root diameter, root volume, specific root length, SPAD values, qP, qL, and ETR (*p* < 0.01); root diameter with root volume, specific root length, SPAD values, qP, qL, and ETR (*p* < 0.01); root volume with specific root length, SPAD values, qP, qL, and ETR (*p* < 0.01); specific root length with SPAD values, qP, qL, and ETR (*p* < 0.01); and SPAD values with qP, qL, and ETR (*p* < 0.01). In addition, SPAD values showed a significant positive correlation with ΦPSII (*p* < 0.05). F_0_ showed a highly significant positive correlation with F_m_ (*p* < 0.01). F_m_ showed a significant positive correlation with F_v_ (*p* < 0.01) and a significant positive correlation with F_v_/F_m_ (*p* < 0.05). F_v_ showed a significant positive correlation with F_v_/F_m_ (*p* < 0.01). ΦPSII showed a significant positive correlation with qL (*p* < 0.01). NPQ showed significant negative correlations with qP, qL, and ETR (*p* < 0.01). qP showed significant positive correlations with qL and ETR (*p* < 0.01), and qL showed a significant positive correlation with ETR (*p* < 0.01). In summary, under Zn stress, plant height, shoot DW, root morphological traits, and SPAD values in *P. chinensis* were mostly significantly positively correlated with photosynthetic activity indicators such as qP, qL, and ETR. At the same time, they were mostly significantly negatively correlated with NPQ.

## 4. Discussion

Zn is an essential micronutrient for plants, yet its biological effects show a distinct concentration dependence. At appropriate concentrations, Zn could participate in enzyme activation and metabolic regulation, whereas excessive Zn can disrupt plant ionic homeostasis and inhibit growth [1,6]. In this study, plant height of *P. chinensis* was significantly reduced at 400 mg L^−1^. In contrast, shoot and root DW and the root-to-shoot ratio showed a numerical decreasing trend but did not differ significantly, indicating that plant height was more sensitive to Zn stress. Li et al. [24] also found in their study on wheat that Zn stress inhibited plant height earlier than it inhibited aboveground biomass. In summary, *P. chinensis* possesses a degree of physiological buffering capacity under short-term Zn stress, and plant height may serve as an early indicator of Zn exposure.

Roots were the first organs of plants to sense Zn stress, and their morphological changes can directly reflect the plant’s degree of adaptation and damage [25]. This study found that total root length and root surface area of *P. chinensis* peaked at 40 mg L^−1^, and root volume peaked at 160 mg L^−1^. Similar phenomena have also been observed in *Arabidopsis thaliana*, where low Zn stress significantly increased total root length, root surface area, and lateral root development [26]. Functionally, this root morphological adjustment may increase the root–substrate contact area and thereby enhance the acquisition of resources such as water and nutrients, consistent with the findings of Morris et al. [27]. At Zn concentrations ≥240 mg L^−1^, total root length, root surface area, and root volume were significantly reduced, indicating that high Zn concentrations inhibited root elongation and the expansion of the absorptive root surface area. Similar phenomena have also been observed in Scots pine and sweet potatoes [28,29]. This inhibition may be associated with disrupted ion homeostasis, reduced root cell elongation and V-ATPase activity, and an altered capacity of root cell walls to bind and immobilize Zn [5,30,31]. In addition, root diameter and specific root length did not change significantly in this study. But excessive Zn increases alfalfa root diameter [32], indicating that different plant species may respond differently to Zn stress in terms of root diameter. The above results indicate that some root traits exhibited concentration dependence. Notably, the inhibition of root morphological traits at 240 mg L^−1^ occurred before any significant reduction in plant height, suggesting that root architecture is a more sensitive endpoint than plant growth.

SPAD values, as an indirect indicator of leaf pigment status, were also highly responsive [33]. In this study, the SPAD values of *P. chinensis* leaves decreased with increasing Zn concentration, and each treatment was significantly lower than that of the CK, indicating that Zn stress may affect the relative chlorophyll content or pigment maintenance capacity of *P. chinensis* leaves. Excessive Zn may impair the photosynthetic capacity of leaves by interfering with the absorption and utilization of Mg and Fe, and by disrupting the stability of pigment-protein complexes [5,7]. Similarly, in *Sinapis alba*, high Zn concentrations reduced photosynthetic pigment levels and impaired photosynthetic performance [34]. In *Ceratoides arborescens*, excess Zn also reduced chlorophyll content and was accompanied by decreases in Fe and Ca concentrations, suggesting that Zn-induced pigment loss may be partly associated with mineral nutrient imbalance [35].

Chlorophyll fluorescence parameters can reflect the operational status of photosystem II (PSII) and the distribution of absorbed light energy in plants [36]. In this study, F_m_, F_v_, F_v_/F_m_, and ΦPSII did not differ significantly among the Zn treatments, suggesting that Zn stress did not markedly impair the maximum photochemical efficiency and the effective quantum yield of PSII, and that PSII function remained relatively stable. Andrejić et al. [7] also found in their study on Miscanthus × Giganteus under Zn stress that although Zn accumulation reduced photosynthetic gas exchange and certain fluorescence parameters, F_v_/F_m_ showed no pronounced decline, suggesting that the maximum quantum efficiency of PSII photochemistry can remain relatively stable in some plant species under Zn stress. Compared with the above indicators, the following ones are more sensitive to zinc stress: qP, qL, and ETR exhibit a decreasing trend as zinc concentration increases, whereas NPQ shows an increasing trend. We also observed a similar phenomenon in *C. arborescens* [35]. qP and qL indicate the degree of openness of PSII reaction centers; ETR reflects the status of electron transport; NPQ represents the plant’s ability to dissipate excess excitation energy via heat-dissipation pathways [36]. These changes indicate reduced PSII reaction center openness and photosynthetic electron transport, together with enhanced thermal energy dissipation in *P. chinensis.* Importantly, these fluorescence responses can be linked directly to the measured tissue Zn concentrations. As shoot Zn concentration increased from 259.2 to 1353.75 mg kg^−1^ DW, NPQ showed a progressive upward trend, indicating that the excess Zn accumulated in leaves enhanced thermal dissipation. This is consistent with NPQ acting as a photoprotective mechanism that dissipates excess excitation energy when Zn interferes with photosynthetic electron transport, as indicated by the concurrent decreases in qP, qL, and ETR. At 400 mg L^−1^, where shoot Zn concentration reached 1353.75 mg kg^−1^ DW, F_0_ decreased significantly, suggesting that a portion of PSII reaction centers may have been damaged or that thylakoid membrane structure was altered. However, the stability of F_m_, F_v_, F_v_/F_m_ and ΦPSII indicates that overall PSII photochemical function was not severely impaired. The significant increase in NPQ indicates that thermal dissipation was substantially enhanced, likely protecting the remaining functional PSII reaction centers by dissipating excess excitation energy. Similar responses have been reported in maize and wheat [37,38]. Therefore, fluorescence parameters exhibited differential sensitivity—F_0_, qP, qL, ETR, and NPQ responded, while F_m_, F_v_, F_v_/F_m_, and ΦPSII remained stable—indicating that regulatory and dissipation processes were already activated before core photochemical efficiency was impaired. At 400 mg L^−1^, a significant decrease in F_0_ and a marked increase in NPQ suggest that PSII in *P. chinensis* may exist in a state where some reaction centers are damaged, overall function is maintained, and protective thermal dissipation is enhanced simultaneously.

Zn accumulation showed a distinct organ-specific pattern. Zn content in the shoots and roots of *P. chinensis* gradually increased with increasing Zn concentration, which was consistent with the results of Repkina et al. in *Sinapis alba* [34]. However, shoot Zn content varied greatly; the maximum value (1353.75 mg kg^−1^ DW) was 5.22 times that of the minimum value (259.2 mg kg^−1^ DW), whereas root Zn content was only 2.96 times greater (maximum 199.48 mg kg^−1^ DW, minimum 67.4 mg kg^−1^ DW). Meanwhile, the highest TF in this study was 7.24, indicating highly efficient root-to-shoot Zn translocation. One of the defining criteria for hyperaccumulator plants is that the metal concentration in shoot must exceed root [14]. The test material satisfies this condition. However, the threshold values for Zn hyperaccumulation are proposed as ≥10,000 mg kg^−1^ DW, and the revised threshold is ≥3000 mg kg^−1^ DW [14,39,40]. In this study, the maximum shoot Zn concentration in *P. chinensis* was 1353.75 mg kg^−1^, which has not yet reached the aforementioned thresholds. Compared to *P. griffithii*, a known Zn hyperaccumulator, this material capacity for Zn accumulation requires further investigation [41]. Furthermore, previous studies have demonstrated that Zn availability in soil cultivation systems was generally lower than that in hydroponic systems [41]. Therefore, our research team will conduct hydroponic experiments in the next phase to further elucidate the maximum Zn accumulation potential in the leaves of *P. chinensis*. It should be emphasized that this study was conducted under controlled conditions, with Zn applied via irrigation solution. The reported Zn concentrations refer to the Zn concentration in the applied irrigation solution rather than the bioavailable Zn concentration in the substrate. Therefore, although *P. chinensis* does not meet the established Zn hyperaccumulation threshold under the current experimental conditions, its high TF and preferential shoot Zn accumulation warrant further investigation; however, its actual performance in soil remediation requires further verification.

## 5. Conclusions

In this study, *P. chinensis* showed responses in SPAD values, certain fluorescence parameters, and root traits to Zn stress earlier than significant changes in plant height, indicating a high translocation efficiency from roots to aboveground parts. When Zn concentration was increased, PSII function was largely maintained, with partial reaction center damage offset by enhanced protective thermal dissipation. It should be emphasized that this study was conducted under controlled conditions, with Zn applied through irrigation solution. The reported Zn concentrations refer to those in the irrigation solution, not the bioavailable Zn concentration in the substrate. Therefore, further validation is needed to assess its practical effectiveness in soil remediation. In summary, Zn accumulation and efficient root-to-shoot translocation were observed in *P. chinensis* under the tested conditions, warranting its further investigation as a candidate species for Zn phytoextraction.

## Figures and Tables

**Table 1:** Effects of Zn stress on growth traits of *Potentilla chinensis*.

Zn Concentration, (mg L^−1^)	Root DW (g)	Shoot DW (g)	Root-to-Shoot Ratio	Plant Height (cm)
CK	0.05 ± 0.00 a	0.19 ± 0.01 a	0.28 ± 0.01 a	13.67 ± 1.18 ab
40	0.05 ± 0.01 a	0.19 ± 0.04 a	0.27 ± 0.03 a	15.23 ± 2.04 a
80	0.06 ± 0.01 a	0.18 ± 0.04 a	0.33 ± 0.11 a	11.90 ± 1.28 bc
160	0.05 ± 0.01 a	0.16 ± 0.01 a	0.29 ± 0.10 a	12.60 ± 0.52 bc
240	0.05 ± 0.01 a	0.15 ± 0.03 a	0.31 ± 0.11 a	11.73 ± 1.06 bc
320	0.05 ± 0.00 a	0.14 ± 0.03 a	0.37 ± 0.11 a	11.27 ± 1.01 bc
400	0.04 ± 0.01 a	0.13 ± 0.03 a	0.31 ± 0.07 a	10.80 ± 1.31 c

Data are presented as mean ± SD (*n* = 3). Different lowercase letters in the same column indicate significant differences among treatments at *p* < 0.05 (ANOVA), whereas values followed by the same letter are not significantly different. CK, control; DW, dry weight.

**Table 2:** Effects of Zn stress on root indices of *Potentilla chinensis*.

Zn Concentration (mg L^−1^)	Total Root Length (cm)	Root Surface Area (cm^2^)	Root Diameter (mm)	Root Volume (cm^3^)	Specific Root Length (cm/g DW)
CK	404.74 ± 1.63 b	23.22 ± 0.19 b	0.15 ± 0.01 ab	0.24 ± 0.01 c	7837.61 ± 202.90 abc
40	426.56 ± 5.87 a	23.81 ± 0.14 a	0.17 ± 0.01 a	0.38 ± 0.09 b	8721.72 ± 1218.70 bc
80	413.37 ± 4.40 b	23.36 ± 0.31 ab	0.16 ± 0.03 ab	0.23 ± 0.03 cd	7573.60 ± 2083.10 abc
160	411.65 ± 1.31 b	23.53 ± 0.08 ab	0.16 ± 0.01 ab	0.46 ± 0.05 a	9657.95 ± 3503.07 c
240	237.98 ± 5.62 c	18.45 ± 0.33 c	0.13 ± 0.02 b	0.17 ± 0.02 de	5391.75 ± 1179.00 a
320	241.23 ± 2.98 c	18.62 ± 0.16 c	0.14 ± 0.01 b	0.13 ± 0.02 ef	4936.52 ± 333.71 a
400	233.65 ± 10.94 c	18.16 ± 0.46 c	0.15 ± 0.01 ab	0.07 ± 0.02 f	5914.09 ± 606.61 ab

Data are presented as mean ± SD (*n* = 3). Different lowercase letters within the same column indicate significant differences among Zn treatments according to one-way ANOVA followed by Duncan’s multiple range test (*p* < 0.05). CK, control treatment.

## Data Availability

All data, tables, and figures in this manuscript are original.
